# Research Progress on Photoperiod Gene Regulation of Heading Date in Rice

**DOI:** 10.3390/cimb46090613

**Published:** 2024-09-16

**Authors:** Jian Song, Liqun Tang, Yongtao Cui, Honghuan Fan, Xueqiang Zhen, Jianjun Wang

**Affiliations:** Institute of Crops and Nuclear Technology Utilization, Zhejiang Academy of Agricultural Sciences, Hangzhou 310021, China; song521125@163.com (J.S.); liquntang2013@126.com (L.T.); cuiyongtao20@163.com (Y.C.); fanhh2024@126.com (H.F.); 15381116236@163.com (X.Z.)

**Keywords:** rice, heading date genes, photoperiod, gene interactions, gene pleiotropy

## Abstract

Heading date is a critical physiological process in rice that is influenced by both genetic and environmental factors. The photoperiodic pathway is a primary regulatory mechanism for rice heading, with key florigen genes *Hd3a* (Heading date 3a) and *RFT1* (RICE FLOWERING LOCUS T1) playing central roles. Upstream regulatory pathways, including *Hd1* and *Ehd1*, also significantly impact this process. This review aims to provide a comprehensive examination of the localization, cloning, and functional roles of photoperiodic pathway-related genes in rice, and to explore the interactions among these genes as well as their pleiotropic effects on heading date. We systematically review recent advancements in the identification and functional analysis of genes involved in the photoperiodic pathway. We also discuss the molecular mechanisms underlying rice heading date variation and highlight the intricate interactions between key regulatory genes. Significant progress has been made in understanding the molecular mechanisms of heading date regulation through the cloning and functional analysis of photoperiod-regulating genes. However, the regulation of heading date remains complex, and many underlying mechanisms are not yet fully elucidated. This review consolidates current knowledge on the photoperiodic regulation of heading date in rice, emphasizing novel findings and gaps in the research. It highlights the need for further exploration of the interactions among flowering-related genes and their response to environmental signals. Despite advances, the full regulatory network of heading date remains unclear. Further research is needed to elucidate the intricate gene interactions, transcriptional and post-transcriptional regulatory mechanisms, and the role of epigenetic factors such as histone methylation in flowering time regulation. This review provides a detailed overview of the current understanding of photoperiodic pathway genes in rice, setting the stage for future research to address existing gaps and improve our knowledge of rice flowering regulation.

## 1. Introduction

Flowering (referred to as heading date) represents the transition of plants from vegetative growth to reproductive growth, serving as the pivotal physiological process determining the reproduction of flowering plants. In rice (*Oryza sativa* L.), the heading date is defined as the time from sowing to when the panicle emerges from the flag leaf, typically indicated when 50% of panicles have emerged. This timing is critical as it affects the period for photosynthetic accumulation, which in turn influences the environmental conditions during the grain filling stage and ultimately impacts rice yield and quality [1]. The heading date of rice is primarily influenced by genetic factors and environmental conditions. Studies in Arabidopsis thaliana have revealed several pathways regulating flowering, including the photoperiodic, circadian clock, vernalization, temperature sensing, autonomous flowering, gibberellin, and age-related pathways. Among these, the photoperiodic pathway plays a pivotal role in rice heading regulation. Understanding the genes involved in this pathway not only sheds light on the molecular mechanisms underlying variation in heading dates, but also offers potential for genetic improvements in rice heading times.

**Figure 1:**
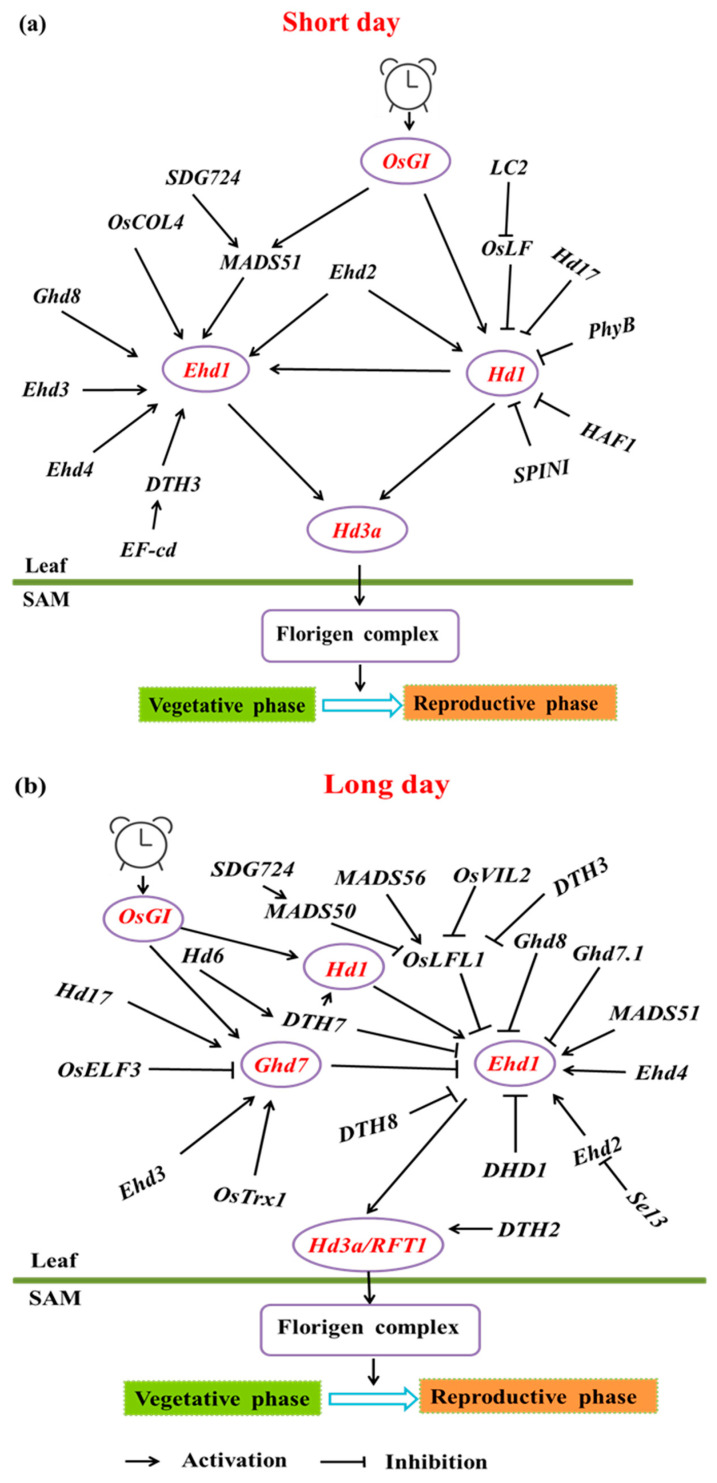
Photoperiodic regulatory pathway of rice flowering. (**a**) Under short-day conditions, *OsGI* activates *Hd1* and *Ehd1*, which promote the expression of the florigen gene *Hd3a*, triggering the transition from the vegetative to the reproductive phase. Various genes, such as *Ghd8*, *OsCOL4*, and *MADS51*, regulate *Ehd1* and *Hd1*, either enhancing or repressing their effects. (**b**) Under long-day conditions, *OsGI* activates *Hd1*, which represses *Hd3a*, while *Ehd1* activates *Hd3a/RFT1* to promote flowering. Other genes, including *Ghd7, DTH7*, and *OsTrx1*, modulate this pathway by controlling *Hd1* and *Ehd1* activity.

Rice is a short-day plant, and different varieties exhibit diverse responses to the photoperiod. Research has identified several key photoperiod-related genes affecting heading date in rice, including *Ehd1*, *Hd1*, *DTH2*, and *OsGI* (Table 1) [2,3,4,5]. Additionally, homologous genes to Arabidopsis flowering genes, such as *RFT1* and *Hd3a*, have been found in rice [6,7,8,9,10]. These genes function similarly to their Arabidopsis counterparts, influencing heading by regulating the apical meristem (SAM) during leaf expression. The photoperiodic induction of heading in rice involves pathways such as *OsGI-Hd1-Hd3a* and *Ghd7-Ehd1-Hd3a/RFT1* (Figure 1). Beyond photoperiodic signals, other factors like gibberellins (GA) and abscisic acid (ABA) also contribute to flowering induction.

This review aims to provide a comprehensive overview of the localization, cloning, and molecular regulatory mechanisms of genes associated with rice heading date. It discusses the current understanding of these genes, their interactions, and their applications in genetic enhancement for optimizing heading date in rice. By highlighting recent advancements and identifying areas requiring further research, this review underscores the importance of these genes in improving rice cultivation and adaptation.

## 2. Photoperiod Gene Regulatory Network for Rice Heading Date

### 2.1. Hd1 and Regulatory Genes

Rice, a model monocot and a typical short-day plant, shares several similarities with Arabidopsis in its response to photoperiod signals, despite some mechanistic differences. Many rice genes homologous to Arabidopsis photoperiod-induced genes have been identified, such as *OsGI*, *Hd1*, *Hd3a*, and *RFT1*. *OsGI* and *Hd1* are homologous to Arabidopsis *GI* and *CO (CONSTANS*), while *Hd3a* and *RFT1* are homologous to the Arabidopsis florigen gene *FT* [7,35]. Additionally, rice heading date regulatory genes *OsMADS14* and *OsMADS15* are homologous to maize *MADS* box gene *ZAP1* [36]. These interactions form a photoperiod regulatory network controlling rice heading date.

*Hd1*, located on chromosome 6, encodes a B-box zinc finger protein and is the first cloned heading date gene in rice [37,38]. Although *Hd1* in rice is highly homologous to *CO* in Arabidopsis, their mechanisms are not entirely identical. In Arabidopsis, *CO* controls flowering by regulating *FT* transcription; under LD conditions, *CO* activates *FT* transcription, promoting flowering; and under SD conditions, *CO* does not affect flowering [39]. In rice, *Hd1* regulates heading date differently: under SD conditions, *Hd1* promotes heading by increasing *Hd3a* expression; under LD conditions, *Hd1* inhibits *Hd3a* transcription, delaying heading. *Hd1* loss-of-function mutants exhibit early flowering under LD conditions [38]. Despite these differences, *Hd1* and *CO* share significant similarities. Both genes are highly conserved in their regulatory pathways: in Arabidopsis, *CO* is regulated by the circadian clock gene *GI*, forming the *GI-CO-FT* pathway. Similarly, in rice, *Hd1* is regulated by the circadian clock gene *OsGI*, forming the *OsGI*-*Hd1*-*Hd3a* pathway [3,20,39,40,41].

*OsGI*, homologous to Arabidopsis *GI* (*GIGANTEA*), is controlled by the circadian clock and inhibits flowering under LD conditions in rice, a characteristic different from Arabidopsis *GI* [42]. *OsGI* antagonizes rice phytochrome genes to regulate *Ghd7* protein stability and flowering time [40]. *HAF1* (*Heading date-associated factor 1*), an E3 ubiquitin ligase with a C3H4 ring domain located on chromosome 4, interacts with *Hd1* and facilitates its degradation via the 26S proteasome pathway. *HAF1* loss-of-function mutants exhibit late heading under both SD and LD conditions. Double mutants for *HAF1* and *Hd1* exhibit heading phenotypes similar to *hd1* mutants under SD conditions and similar to *haf1* mutants under LD conditions, indicating that *HAF1* affects heading primarily through *Hd1* transcriptional activity under SD conditions [43]. *OsELF3*, a direct substrate of *HAF1*, is involved in circadian regulation and photoperiod-induced heading. Double mutants for *HAF1* and *OsELF3* show heading times similar to *oself3* plants under LD conditions [32]. *SPIN1* (*SPL11-interacting protein 1*) interacts with the E3 ubiquitin ligase *SPL11* and acts as a repressor of rice heading: under SD conditions, *SPIN1* delays heading by mediating *Hd1* inhibition of *Hd3a* transcription; under LD conditions, *SPIN1* regulates heading independently of *Hd1* [44]. *RBS1* (*RNA-binding and SPIN1-interacting 1*) interacts with *SPIN1* to regulate heading, inhibiting heading under both SD and LD conditions. *RBS1* activates *SPIN1* transcription while inhibiting *SPL11* expression [45].

The rice heading gene *Hd6* encodes the α subunit of protein kinase CK2 (CK2α) and delays heading under LD conditions by inhibiting *FT-like* gene expression [35]. *Hd6* regulation of heading is controlled by *Hd1*, which modulates *Hd6* transcriptional activity based on the photoperiod [46]. *SE5* (*PHOTOPERIOD SENSITIVITY5*) senses light signals by regulating *Hd1* transcription [47]. *SE5* loss-of-function mutants exhibit early heading under both SD and LD conditions due to altered light signal pathways, making *Hd1* insensitive to light [7,48]. Photoperiod sensitivity in rice heading involves major genes such as *Hd1*, *Ghd7*, and *DTH8*. *Hd1* promotes heading under all photoperiods when *Ghd7* and *DTH8* are knocked out, while *Ghd7* inhibits heading. Under LD conditions, *Hd1* promotes *Ghd7* expression, and *Ghd7* and *DTH8* form inhibitory complexes that partially or completely silence the *Ehd1*-*Hd3a*/*RFT1* pathway, leading to delayed or absent heading. Under SD conditions, low *Ghd7* expression allows *Hd1* to compete with the inhibitory complex, promoting *Hd3a*/*RFT1* expression and heading [3]. Therefore, rice photoperiodic flowering is controlled by two interacting modules: the *Hd1*-*Hd3a*/*RFT1* pathway under SD and the (*Hd1*/*Ghd7*/*DTH8*)-*Ehd1*-*Hd3a*/*RFT1* pathway under LD. These genetic differences provide a basis for the wide adaptability of rice.

### 2.2. Ehd1 and Regulatory Genes

*Ehd1* is a critical regulator of rice heading date, located on chromosome 10, which encodes a B-type response regulator unique to rice. Under SD conditions, *Ehd1* can promote heading independently of *Hd1*, indicating that *Ehd1’s* regulation of heading does not rely on *Hd1*. Moreover, *Ehd1* promotes heading under both LD and SD conditions, differing from *Hd1* in its response to photoperiod signals. Currently, no direct homologs of *Ehd1* have been identified in the Arabidopsis genome. Under SD conditions, *Ehd1* promotes rice heading by inducing the expression of *Hd3a* and *RFT1* [49]. Extensive research has been conducted on *Ehd1*-mediated heading in rice. *Ehd1* interacts with an A-type response regulator, *OsRR1*, forming a heterodimer. Overexpression of *OsRR1* results in a late flowering phenotype, suggesting that *OsRR1* may inhibit *Ehd1* transcription, thereby affecting heading [50]. Cytokinins can induce the expression of *OsRR1* and *OsRR2*, which bind to *Ehd1* to form inactive complexes, suppressing *Ehd1’s* transcriptional activity. This indicates that exogenous cytokinins can delay flowering by inhibiting *Ehd1*, thereby prolonging the vegetative growth period [2]. While *Ehd1* determines heading by regulating the transcription of *Hd3a* and *RFT1*, the interaction mechanisms among *Ehd1*, *Hd3a*, and *RFT1* vary across different rice varieties. Studies involving segregating populations with various allelic combinations of *Ehd1*, *Hd3a*, and *RFT1* revealed that *Hd3a* remains silent when *Ehd1* is mutated, but *RFT1* still exhibits transcriptional activity, albeit at a lower level compared to plants with functional *Ehd1*. Additionally, a null allele of *RFT1* (*rft1*) was identified in these populations. Plants with homozygous mutations in *ehd1* and *hd3a*/*rft1* do not induce heading. Similar to *Hd3a*, *RFT1* can interact with the florigen receptor 14-3-3 protein, but *RFT1* null mutants cannot. Furthermore, research on sequence variations and geographical distribution indicates that functional *RFT1* alleles were selected during the adaptation of rice to high-latitude regions [51].

*Ehd2*/*RID1*/*OsId1*/*Ghd10* is a critical factor in rice flowering transition, encoding a zinc finger transcription factor homologous to the maize flowering promoter *ID1* (*INDETERMINATE1*). In *ehd2* mutants, *Hd1* is insensitive to photoperiod, showing a late heading phenotype under both LD and SD conditions, indicating that *Ehd2* is crucial for heading transition. In *ehd2* mutants, the transcription levels of *Ehd1*, *Hd3a*, and *RFT1* are significantly down regulated under both long and short-day conditions, suggesting that *Ehd2* influences heading primarily by promoting the transcriptional activities of *Ehd1*, *Hd3a*, and *RFT1* [16]. *RID1* can directly bind to the promoters of *Hd3a* and *RFT1*, activating their expression and maintaining the chromatin accessibility at their transcription start sites through H3K4me3, H3K9ac, and H3K36me3 deposition, thereby promoting flowering [17].

The *Ehd3* gene encodes a transcription factor with two PHD-type zinc fingers. Under LD conditions, *ehd3* mutants exhibit delayed heading compared to the wild type. In *ehd3* mutants, *Ghd7* transcription is activated, suppressing the expression of *Ehd1*, *Hd3a*, and *RFT1*, indicating that *Ehd3* acts as a promoter of *Ehd1* in the rice heading regulatory network [16]. *Ehd4* encodes a new CCH-type zinc finger protein and acts as a positive regulator of *Ehd1*. *Ehd4* loss-of-function mutants (*ehd4*) do not head under LD conditions. The expression pattern of *Ehd4* in rice is similar to that of *Ehd1*, and it regulates the transcription of *Hd3a* and *RFT1* through *Ehd1* [18].

*OsCOL4* (*CONSTANS*-like) loss-of-function mutants (*oscol4*) flower early under both LD and SD conditions, whereas overexpression of *OsCOL4* results in late flowering under both conditions. In *oscol4* mutants, the transcriptional activities of *Ehd1*, *Hd3a*, and *RFT1* are increased, while overexpression of *OsCOL4* reduces their transcription, indicating that *OsCOL4* acts upstream of *Ehd1* as a heading repressor [34].

*OsMADS51* is a SD heading promoter. *OsMADS51* loss-of-function mutants flower 15 days later than wild-type under SD conditions, with minimal impact under LD conditions. In these mutants, the expression levels of *Ehd1*, *OsMADS14*, and *Hd3a* are significantly reduced. Overexpression of *OsMADS51* leads to earlier heading under short-day conditions and upregulation of *Ehd1*, *OsMADS14*, and *Hd3a*. These results suggest that *OsMADS51* is a SD heading promoter, acting upstream of *Ehd1*, *OsMADS14*, and *Hd3a*. Furthermore, *OsMADS51* expression is not affected in *Ehd1* RNAi lines, confirming that *OsMADS51* acts upstream of *Ehd1* [52]. Co-expression of *OsMADS51* with *OsFLZ2* destabilizes *OsMADS51*, weakening its transcriptional activation of downstream target genes like *Ehd1* [53].

*Ehd5*, encoding a WD40 domain-containing protein, is light-inducible and exhibits a circadian rhythm in its expression pattern. Transcriptome analysis has identified *Ehd5* as an upstream regulator of flowering genes *Ehd1*, *RFT1*, and *Hd3a* [54]. OsCIBL1 interacts with OsCRY2, a member of the rice CRY family (OsCRY1a, OsCRY1b, and OsCRY2), and binds to the *Ehd1* promoter, thereby activating the rice-specific *Ehd1*-*Hd3a*/*RFT1* pathway for flowering induction [55]. *OsWRKY11* facilitates the formation of a ternary protein complex involving *OsWRKY11*, *Hd1*, and *DTH8*; this complex subsequently suppresses *Ehd1* expression, leading to a delayed heading date [56].

### 2.3. Ghd7 and Regulatory Genes

*Ghd7* is associated with traits such as grain number per panicle, plant height, and heading date in rice. It encodes a nuclear protein with a CCT domain. Under long-day conditions, overexpression of *Ghd7* results in significantly delayed heading, increased plant height, and larger panicle size compared to wild-type plants [19]. *Ghd7* is induced by phytochromes and suppresses the transcription of *Ehd1*. The interaction between the heading promoter *Ehd1* and the repressor *Ghd7* regulates the transcriptional activity of *Hd3a*, thereby affecting heading [57].

Overexpression of *OsMFT1* delays heading by approximately seven days compared to the wild type, and significantly increases the number of spikelets per panicle and branches. In contrast, *OsMFT1* knockout mutants show earlier heading and reduced spikelet numbers. Overexpression of *OsMFT1* significantly suppresses *Ehd1* transcription while activating *Ghd7* expression. Additionally, the transcription factor *OsLFL1* directly binds to the RY element in the *OsMFT1* promoter, activating its transcription. This indicates that *OsMFT1* is a heading repressor, acting downstream of *Ghd7* and upstream of *Ehd1*, primarily regulating rice heading at the transcriptional level [31].

Variations in the alleles of *Hd1*, *Ghd7*, and *DTH8* lead to differences in photoperiod sensitivity among rice populations. Studies indicate that rice photoperiod-sensitive flowering is controlled by the *Hd1*-*Hd3a*/*RFT1* module under SD conditions and the (*Hd1*/*Ghd7*/*DTH8*)-*Ehd1*-*Hd3a*/*RFT1* module under LD conditions. These genetic differences provide the basis for rice’s wide environmental adaptability. The *Ghd8*-*OsHAP5C*-*Ghd7* triple complex directly binds to the *Hd3a* promoter, downregulating the expression of *Ehd1*, *Hd3a*, and *RFT1*, ultimately leading to delayed heading [21]. *OsCOL5*, an ortholog of Arabidopsis *COL5*, is involved in photoperiodic flowering and enhances rice yield through modulation of *Ghd7* and *Ehd2* and interactions with *OsELF3*-1 and *OsELF3*-2 [58]. *OsCOL10* encodes a member of the CONSTANS-like (COL) family, which represses the expression of the *FT-like* genes *RFT1* and *Hd3a* through *Ehd1.* Transcripts of *OsCOL10* are more abundant in plants carrying a functional Gh*d*7 allele or overexpressing *Ghd7* than in *Ghd7*-deficient plants, thus placing *OsCOL10* downstream of *Ghd7* [59].

## 3. Gene Interaction Regulates Rice Heading Together

Gene interactions during the heading stage of rice have elucidated synergistic effects and signaling mechanisms among multiple genes involved in the regulation of rice flowering. These investigations advance our understanding of the complex regulatory networks that govern rice growth and development, while also laying the groundwork for theoretical advancements in breeding new varieties with enhanced agronomic traits [20,60]. The photosensitivity of rice at the heading stage involves several key genes, including *Hd1*, *Ghd7*, and *DTH8*. Irrespective of day length, the single knockout of *Hd1* (*Ghd7* and *DTH8*) promotes heading, whereas single *Ghd7* inhibits it. Under LD conditions, *Hd1* enhances *Ghd7* expression and forms distinct inhibitory complexes with *Ghd7* and *DTH8*, thereby partially suppressing (double knockout) or completely silencing (triple knockout) the *Ehd1*-*Hd3a*/*RFT1* flowering pathway, resulting in varying degrees of delayed or absent heading. Conversely, under SD conditions, *Ghd7* expression is significantly reduced, leading to a competitive relationship between *Hd1* and the inhibitory complex. This promotes *Hd3a*/*RFT1* expression to varying degrees, thereby influencing heading. Thus, photoperiodic flowering in rice is governed by two modules: *HD1*-*HD3A*/*RFT1* under SD conditions and (*Hd1*/*Ghd7*/*DTH8*)-*Ehd1*-*Hd3a*/*RFT1* under LD conditions. Variations in these genes provide the foundation for broad adaptability in rice [3]. The genetic interaction of four major genes *Ghd7*, *Ghd8*, *OsPRR37*/*Ghd7.1* and *Hd1* in the rice heading stage was analyzed. The four genes had four-gene, three-gene, and gene interactions in both conditions, but were more significant in NLD (natural long-day) conditions. In the context of functional *Hd1*, *Ghd7* had the strongest gene interaction with *Ghd8* under NLD conditions, while *Ghd7* had the strongest gene interaction with *PRR37* under NSD (natural short-day) conditions. Interestingly, *PRR37* acts as a flowering suppressor under NLD conditions, while under NSD conditions, it can alternately act as an activator or suppressor depending on the state of the other three genes [61]. *OsCOL5*, a homologue of Arabidopsis *COL5*, is involved in photoperiodic flowering and increases rice yield by regulating *Ghd7* and *Ehd2* and interacting with *OsELF3-1* and *OsELF3-2* [58]. *OsCOL13* negatively regulates the flowering of rice under both long- and short-day conditions and inhibits the floral genes *Hd3a* and *RFT1* by inhibiting *Ehd1* [62].

Histone haploidin-1 (OsHUB1) and OsHUB2 are involved in the regulation of heading date through *Hd1* and *Ehd1* pathways. In both LD and SD conditions, the loss of *OsHUB1* and *OsHUB2* function resulted in early heading dates. The expression of *Hd3a*, *RFT1*, and *Ehd1* was induced under LD conditions, and the transcription levels of *Hd1*, *Ghd7*, *OsCCA1*, *OsGI*, *OsFKF1*, and *OsTOC1* were decreased, while the expression of *RFT1* and *Ehd1* was induced by the *oshub2* mutant under SD conditions [63]. *OsFTL12* is an important factor in the regulation of rice heading, and it has antagonistic effects on *Hd3a* and *RFT1*. Unlike *Hd3a* and *RFT1*, *OsFTL12* is not regulated by day length and is highly expressed under both SD and LD conditions, and the heading date is delayed under both SD and LD conditions. OsFTL12 interacts with GF14b and OsFD1, two key components of the anthocyanogen activation complex (FAC), and forms the anthocyanogen inhibition complex (FRC) by competitively binding to GF14b with *Hd3a*. In addition, OsFTL12-FRC can bind the promoters of the floral identity genes *OsMADS14* and *OsMADS15* and inhibit the expression of both [64]. *OsFLZ2* is a negative regulator at heading stage of rice. It interacts with *OsMADS51* and disrupts the stability of *OsMADS51*, weakening its transcriptive activation of downstream target gene *Ehd1* [53]. Therefore, by identifying and utilizing the gene interaction at heading stage, the flowering time and growth period of rice can be accurately regulated, and the yield stability and adaptability can be improved to meet the growing global food demand.

## 4. Pleiotropy of Rice Heading Date Genes

Recently, with the advancing research on the functional mechanisms of rice heading date genes, a series of pleiotropic genes have been discovered. These genes exhibit a phenomenon known as one gene, multiple effects, where a single gene or gene pair on the chromosome influences multiple phenotypic traits. For example, *Ehd1* functions as a pivotal integrative gene in the regulatory network controlling rice heading, with its expression levels negatively correlated with heading date and yield [65]. Using CRISPR-Cas9 technology, the *Ehd1* gene was edited in the elite Northeast japonica rice varieties Jiyuanxiang 1 and Yinongxiang 12. Field trials showed that these new *ehd1* mutants not only outperformed the wild type in yield under low latitude conditions, but also retained their superior agronomic traits [66]. Both the *Ehd1* and the *Hd1* genes reduce the number of primary branches in the panicle, resulting in a decreased grain number. These important flowering genes likely regulate rice panicle development by affecting the expression of florigen genes in the leaves, thereby influencing crop yield in the field [67]. *DTH7*/*Ghd7*.1/*OsPRR37*/*Hd2* represents a major genetic locus controlling rice photoperiod sensitivity and grain yield, encoding a pseudo-response regulator protein whose expression is regulated by photoperiod [23,68]. *DTH8*/*Ghd8*/*LHD1*, encoding the HAP3H subunit of the transcription factor CCAAT-box binding protein, has been shown to concurrently regulate rice yield, plant height, and heading date [61,69]. Overexpression of *DHD1* delays rice heading and enhances agronomic traits such as panicle length, tiller number, and grain number, thereby increasing yield [70]. *Ghd7* and *PRR37* promote the spikelet number and yield, with a synergistic effect observed in *Ghd7*–*DTH8* or *Ghd7*–*PRR37* combinations, although *Hd1* negatively impacts yield [71]. *OsMFT1* acts as a flowering inhibitor, operating downstream of *Ghd7* and upstream of *Ehd1*, while also positively regulating rice panicle morphology [31].

The efficiency of nitrogen utilization in plants is primarily regulated by genetic factors, environmental influences, and their interactions. The *ARE1* gene serves as a key regulator of nitrogen nutrition and yield. Studies have revealed that *Ghd7* binds to the *ARE1* gene and suppresses its expression, thereby positively regulating nitrogen utilization and yield in rice. It is noteworthy that the Gh*d*7 and *ARE1* haplotypes are closely associated with soil nitrogen content and exhibit differential distribution patterns among rice subpopulations, making them selected loci in rice breeding processes. Furthermore, the combination of superior alleles from both genes enhances nitrogen use efficiency and yield under low nitrogen conditions, offering genetic markers for breeding nitrogen-efficient materials [72].

*Ghd7* regulates the balance between abscisic acid (ABA) and gibberellins (GAs), increasing the ABA/GA3 ratio to inhibit seed germination. During seed germination, the mutant *ghd7* exhibits reduced sensitivity to exogenous ABA, leading to decreased ABA accumulation in mature *ghd7* seeds due to diminished *OsNCED* gene expression. Additionally, elevated GA3 levels during seed imbibition in *ghd7* seeds are attributed to induction of genes involved in biologically active GA synthesis pathways [73]. Overexpression of *OsABF1* in plants results in a late-flowering phenotype. Simultaneous RNAi knockdown of *OsABF1* and its most homologous gene, *OsbZIP40*, causes a significant early-flowering phenotype. Moreover, overexpression of *OsABF1* leads to a typical gibberellin-deficient phenotype, semi-dwarfism, and delayed seed germination [74,75,76]. During rice heading, the gene *OsSOC1*/*OsMADS50*/*DTH3* regulates the number of adventitious roots in rice. *OsMADS50* acts as a target gene of *OsSPL3* or *OsSPL12*, which are targets of *Osmir156*. Thus, the “*miR156*-*OsSPL3*/*OsSPL12*-*OsMADS50*” module constitutes a regulatory pathway involved in rice adventitious root formation through modulation of auxin transport and signaling [77].

## 5. Prospects

Research on rice flowering regulation has significantly expanded our understanding of the molecular mechanisms of flowering induction and regulation. As new genes are discovered and the mechanisms of gene interactions are further understood, the molecular regulatory network is becoming increasingly complex. The molecular network for flowering regulation involves hormone pathways, autonomous pathways, and temperature pathways, which interact with the photoperiod pathway to precisely control flowering time, highlighting the complexity of flowering mechanisms.

Despite significant progress in the *Hd1* and *Ehd1* regulatory pathways, with numerous related genes cloned, the interaction mechanisms among these genes and their responses to environmental signals, as well as the transcriptional and post-transcriptional regulation of *Hd1* and *Ehd1*, remain unclear. The role of histone methylation proteins in the epigenetic regulation of flowering time, such as *SDG708*, *SDG724*, *SDG725*, *OsTrx1*, and *OsVIL2*, also requires further investigation. With advancements in genome sequencing, transcriptome analysis, CRISPR gene editing, and large-scale screening of interacting proteins, new flowering genes and genetic regulatory networks will continue to be discovered. This research will provide valuable insights into the ecological and environmental survival strategies of different species.

The study of rice flowering regulation mechanisms has broad applications beyond basic research. Research on pivotal flowering genes such as *Hd3a*, *DTH8*, and *Ghd7* has illustrated heterosis, with different allele combinations outperforming parental lines in traits like grain number per panicle, seed setting rate, and grain yield. These discoveries are widely applied in hybrid rice breeding. Selecting optimal allele combinations of flowering genes for specific regions from low to high latitudes will provide breeders with effective choices for developing eco-type varieties and lay the foundation for future molecular design breeding.

## 6. Conclusions

In summary, the regulation of rice heading dates through photoperiodic genes involves a complex network of interactions that govern the timing of flowering. The identification and functional analysis of key genes such as Hd1, Ehd1, Ghd7, and their associated pathways have provided significant insights into the molecular mechanisms that control this crucial developmental stage in rice. The interplay between these genes and their responses to environmental signals, such as day length, highlights the intricate balance required for optimal flowering and, consequently, rice yield.

While much progress has been made, our understanding of the photoperiodic regulation of heading date is still incomplete. Many of the regulatory interactions remain to be fully elucidated, particularly in the context of varying environmental conditions and different rice cultivars. Further research is needed to explore these aspects, which will not only advance our fundamental knowledge of plant biology but also have practical implications for breeding programs aimed at improving rice’s adaptability and productivity under diverse agricultural conditions.

This review consolidates current knowledge on the photoperiodic control of rice heading date and points to future directions for research, emphasizing the need for a more comprehensive understanding of gene interactions and the potential for genetic manipulation to optimize rice cultivation.

## Figures and Tables

**Table 1:** Key genes involved in the photoperiodic regulation of flowering in rice.

Gene	Gene ID	Primary Function	References
*Hd3a/FTL2*	LOC_Os06g06320	Promotes flowering; acts as a florigen gene under SD (short-day) conditions.	[9,10]
*RFT1/FTL3*	LOC_Os06g06300	Promotes flowering; acts as a florigen gene under LD (long-day) constitutive flowering repressor conditions.	[11]
*OsGI*	LOC_Os01g08700	Circadian rhythm gene; activator of *Hd1*; promotes flowering under SD and inhibits under LD.	[4,12]
*Hd1*	LOC_Os06g16370	Encodes a zinc finger protein with 395 amino acids; promotes flowering under SD and inhibits under LD; a key integrator in the *OsGI*-*Hd1*-*Hd3a* pathway.	[13,14]
*Ehd1*	LOC_Os10g32600	Early heading quantitative trait locus (QTL) derived from African cultivated rice (*Oryza glaberrima* Steud.); promotes heading under SD conditions independently of *Hd1.*	[2,15]
*Ehd2/RID1/OsId1/Ghd10*	LOC_Os10g28330	Encodes a zinc finger transcription factor; promotes heading and initiates flowering induction.	[16,17]
*Ehd3*	LOC_Os08g01420	Promotes flowering; encodes a PHD-type zinc finger protein; induces heading by suppressing *Ghd7* or upregulating *Ehd1* under LD.	[16]
*Ehd4*	LOC_Os03g02160	Promotes flowering; encodes a CCH-type zinc finger protein; upregulates florigen genes via *Ehd1,* without direct binding to the *Ehd1* promoter region.	[18]
*Ghd7/Hd4/E1*	LOC_Os07g15770	A major QTL controlling panicle number per plant, plant height, and heading date.	[19,20]
*DTH8/Ghd8/LHD1/OsHAP3H/EF8/OsNF-YB11/CAR/LH2*	LOC_Os08g07740	Encodes a polypeptide with 297 amino acids; delays flowering under LD by regulating *Ehd1*, *RFT1*, and *Hd3a*; promotes flowering under SD.	[13,21]
*DTH7/Ghd7.1/OsPRR37/Hd2*	LOC_Os07g49460	Major locus controlling photoperiod sensitivity and grain yield; encodes a pseudo-response regulator protein regulated by photoperiod.	[22,23]
*OsCO3*	LOC_Os09g06464	Flowering inhibitor; regulates flowering time mainly under SD, independent of the SD florigen pathway.	[5,24]
*OsPhyA*	LOC_Os03g51030	Affects heading date by regulating *OsGI* under SD and *Ghd7* under LD.	[25]
*OsPhyB*	LOC_Os03g19590	Regulates *Hd1*-mediated expression of florigen *Hd3a* and critical day length.	[26,27]
*OsELF3/Hd17/Ef7*	LOC_Os01g38530	Circadian gene; promotes flowering under LD.	[14,28]
*Hd6/CK2α*	LOC_Os03g55389	Delays flowering under LD by inhibiting the expression of *FT-like* genes.	[29]
*OsLFL1*	LOC_Os01g51610	Encodes a transcription factor with a B3 domain; regulates heading date by directly binding to the *OsMFT1* promoter through the RY motif.	[30,31]
*HAF1*	LOC_Os04g55510	E3 ubiquitin ligase with a C3H4 ring domain; regulates heading date through interaction with *Hd1.*	[32]
*OsCOL4*	LOC_Os02g39710	Constitutive flowering repressor in rice; acts upstream of *Ehd1* and downstream of *OsphyB.*	[33,34]
